# Hydrodynamic Cavitation in Juice Processing: Linking PME, PPO, and POD Responses to Physicochemical Stability and Quality Retention

**DOI:** 10.3390/molecules31183323

**Published:** 2026-09-19

**Authors:** Lorenzo Albanese

**Affiliations:** Institute of BioEconomy, National Research Council of Italy, Via Madonna del Piano 10, 50019 Florence, Italy; lorenzo.albanese@cnr.it

**Keywords:** hydrodynamic cavitation, pectin methylesterase, polyphenol oxidase, peroxidase, juice processing, matrix specificity, enzyme–property linkage, physicochemical stability, quality retention, process attribution

## Abstract

Hydrodynamic cavitation (HC) is increasingly investigated for juice processing, but changes in pectin methylesterase (PME), polyphenol oxidase (PPO), and peroxidase (POD) activity do not by themselves establish a technological benefit. Several mild or temperature-limited conditions left substantial residual activity, whereas stronger control was observed in thermally assisted, more severe, or hurdle-assisted treatments. Physical stability can nevertheless improve despite limited PME or PPO inactivation, consistent with concurrent particle-size reduction, pectin restructuring, rheological modification, and other matrix-level changes. Within-study quantitative comparisons showed that, in all six directly comparable enzyme-level contrasts, inactivation at the selected or experimentally validated condition was lower than the largest directly observed value for the same enzyme. In the three exact same-condition PPO storage trajectories, end-of-storage inactivation was lower than at day 0. Current evidence therefore supports treatment-level responses more strongly than cavitation-specific causality and does not justify a universal HC operating window. Technologically meaningful process development requires matrix-, enzyme-, and reactor-specific conditions that provide sufficient enzyme control for a defined product function, a favorable linked physicochemical response, acceptable quality retention, and persistence during storage, supported by adequate hydraulic and thermal characterization and controls matched to the causal claim.

## 1. Introduction

Endogenous enzymes remain a major processing constraint in juice stabilization because their technological relevance is expressed through specific product functions rather than enzyme activity alone. Pectin methylesterase (PME) modifies the degree of methylesterification of pectin and can promote destabilization of the dispersed cloud phase, whereas polyphenol oxidase (PPO) and peroxidase (POD) contribute through distinct oxidative pathways to browning, pigment modification, and the transformation or loss of phenolic compounds [1,2,3]. The technological significance of an enzyme response therefore depends on whether it is accompanied by a favorable response in the property that the enzyme is expected to influence, either immediately after processing or during subsequent storage. This relationship is especially important in juices, where cloud stability, sedimentation, color, rheological behavior, sensory attributes, and retention of heat- or oxidation-sensitive compounds can simultaneously determine product quality.

Hydrodynamic cavitation (HC) has consequently attracted growing interest as a process-intensification route for liquid foods. Cavitation develops when flow through a restriction produces a sufficient local pressure decrease for vapor cavities to form, followed by their collapse as pressure recovers. The process environment experienced by the product, however, is not defined by cavity collapse alone. Reactor geometry, pressure drop, flow rate, recirculation, turbulence, shear, and temperature jointly determine the treatment state, and cavitation number cannot be used as a stand-alone severity descriptor transferable across different devices or operating conditions [4,5,6]. Hydrodynamic stresses coexist with cavitation, while temperature can influence both enzyme-inactivation kinetics and cavitation intensity or regime [7,8]. HC should therefore not be classified a priori as intrinsically non-thermal, and observed treatment responses should not be assigned specifically to cavitation when the accompanying thermal and hydraulic contributions have not been adequately separated.

The relevant technological problem is consequently more complex than maximizing enzyme inactivation. Mild conditions may better preserve nutrients and sensory properties while leaving residual enzyme activity too high for the required product function; greater severity may strengthen enzyme control while increasing thermal or oxidative burden. Evidence from other juice-stabilization technologies likewise shows that resistant enzyme fractions, temperature, and treatment combinations can determine both the magnitude and persistence of inactivation [9,10,11]. In HC, interpretation is further complicated by direct changes in particle size, pectin organization, rheology, and dispersed-phase structure. Physical stability may therefore improve despite substantial residual enzyme activity, whereas stronger inactivation may be accompanied by quality losses that reduce its technological value. Enzyme control and quality retention must thus be assessed together rather than treated as independent outcomes.

Reviews have already addressed cavitation principles, reactor configurations, operating parameters, and general food-processing applications of HC [12,13,14]. Physicochemical modification, fresh-produce beverages, and engineering aspects of liquid-food processing have also been examined directly [15,16,17]. It remains insufficiently resolved whether PME, PPO, and POD responses reported in real juices are accompanied by favorable changes in the physicochemical property most directly associated with each enzyme; whether that product-level response remains favorable when quality retention and storage persistence are considered; and to what extent the observed response can be distinguished from concomitant effects of temperature, recirculation, hydrodynamic stresses, homogenization, and formulation. 

**Figure 1:**
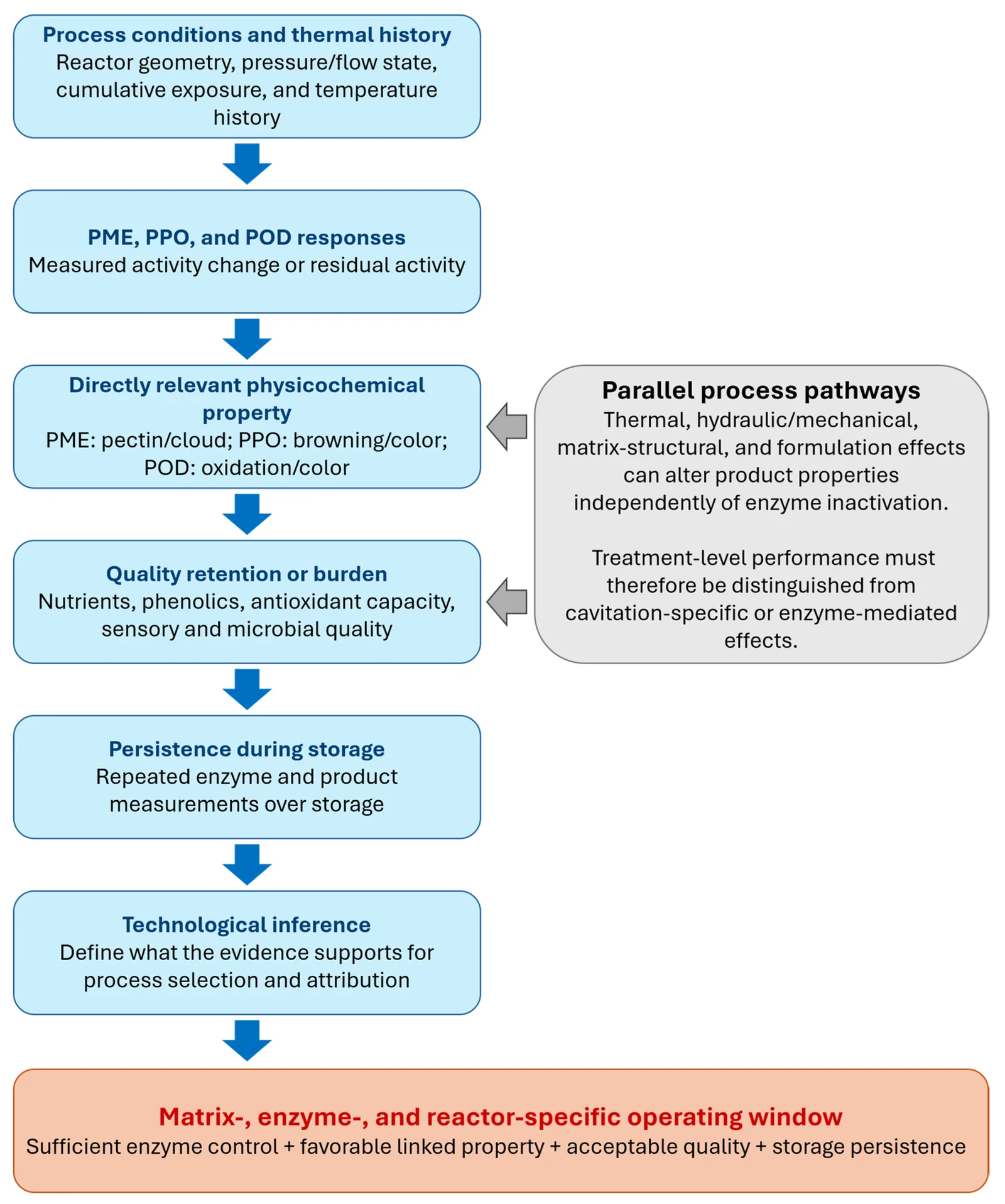
Interpretive framework linking HC process conditions, enzyme responses, product properties, quality retention, storage persistence, and technological inference in juice processing.

The central question is therefore not whether HC can modify PME, PPO, POD, or juice physicochemical properties individually, but under which conditions an enzyme response is linked to the relevant product function, remains compatible with quality retention and storage persistence, and can be attributed at the level supported by the experimental design. A defensible assessment requires the interpretive sequence summarized in Figure 1: process conditions and thermal history → enzyme response → directly relevant physicochemical property → quality retention or burden → persistence during storage → technological inference. Parallel thermal, hydraulic/mechanical, matrix-structural, and formulation pathways can modify product properties independently of enzyme inactivation. This distinction separates enzyme control from treatment-induced physical or compositional changes and prevents the performance of the complete HC-based process from being interpreted automatically as evidence of cavitation-specific or enzyme-mediated causality. The resulting framework provides a basis for defining matrix-, enzyme-, and reactor-specific operating windows in which sufficient enzyme control is accompanied by a favorable linked property, acceptable quality retention, and persistence during storage.

As a simplified example, a decrease in PME activity accompanied by improved cloud stability would satisfy the enzyme-response and directly relevant physicochemical-property steps in Figure 1. If particle-size reduction, pectin restructuring, or other parallel process effects occur concurrently, however, the improved physical stability cannot be attributed to PME inactivation alone. Evidence of durable enzyme control would additionally require repeated PME measurements together with the associated physical-stability response during storage. 

The framework in Figure 1 complements rather than replaces conventional thermodynamic descriptions of the processing state and enzyme-inactivation kinetic models. Thermodynamic variables such as pressure and temperature define key components of the physical treatment state, whereas kinetic models relate changes in enzyme activity to processing exposure under specified conditions [4,5,9]. Within Figure 1, these established descriptions correspond primarily to the first two steps: process conditions and thermal history, and PME, PPO, and POD response. The framework then extends the interpretation beyond those levels by requiring evidence that the enzyme response is associated with the directly relevant physicochemical property, remains compatible with acceptable product quality, and persists during storage. This extension is particularly important for HC because hydraulic/mechanical and thermal contributions coexist within the treatment state [7,8]. Consequently, successful description or prediction of enzyme inactivation under a defined HC condition does not, by itself, establish enzyme-mediated product improvement or cavitation-specific causality. Figure 1 therefore provides an inferential bridge from process-state and kinetic descriptions to product function, quality burden, storage persistence, and the level of technological attribution supported by the experimental design.

## 2. Scope and Literature Search

The literature search was conducted in Scopus, Web of Science, and PubMed using the title, abstract, and keyword fields available in each database. The initial search used the combination “hydrodynamic cavitation” AND juice. Subsequent searches used “hydrodynamic cavitation” AND juice AND (“pectin methylesterase” OR “pectin methyl esterase” OR PME), “hydrodynamic cavitation” AND juice AND (“polyphenol oxidase” OR PPO), and “hydrodynamic cavitation” AND juice AND (peroxidase OR POD). The searches were supplemented by citation tracking and updated through August 2026. No language filter was applied during the database searches.

Search results were screened, and peer-reviewed experimental studies were included when hydrodynamic cavitation was applied directly to a real fruit or vegetable juice, at least one experimental measurement of PME, PPO, or POD was reported, and the processing conditions were sufficiently described to interpret the enzyme response. Studies involving non-juice matrices, non-experimental publications, studies without measurements of the target enzymes, and treatment designs in which the contribution of HC could not be interpreted within the defined scope were excluded. Eligibility was verified from the full text. For each included study, the full article and, when available, the associated supplementary materials were examined. Within the defined scope, 11 primary studies met the inclusion criteria.

Quantitative results were used when explicitly reported in the source, with experimentally measured values kept distinct from model-derived predictions. Exact quantitative comparisons were restricted to unambiguous within-study, same-enzyme values. PME, PPO, and POD were considered separately in relation to the directly relevant product property, quality retention, and storage behavior when available.

## 3. Enzyme–Quality Relationships in Juice Matrices

### 3.1. Pectin Methylesterase

Pectin methylesterase (PME) catalyzes the de-esterification of methyl-esterified galacturonic acid residues in pectin, decreasing the degree of methylesterification and increasing the number of free carboxyl groups. In citrus juices, the resulting pectin can interact more readily with calcium and other cloud constituents, favoring flocculation and loss of dispersed-phase stability. The technological significance of PME is therefore expressed through changes in pectin functionality and cloud behavior rather than through enzyme activity alone. Orange-juice pectinesterase fractions also differ in thermal stability and cloud-destabilizing capacity, so the effect of residual PME depends on the enzyme population and matrix rather than on a single generic activity threshold [1,18]. Direct inhibition of citrus PME with a purified pectin methylesterase inhibitor correspondingly maintained relative turbidity, viscosity, pH, and ζ-potential, while limiting sedimentation [19]. PME-related instability is most directly reflected by pectin state, cloud retention, turbidity, sedimentation, and rheological behavior. These properties, however, are also controlled by particle-size distribution, pectin molecular architecture, serum-phase composition, and interactions among suspended solids and soluble polymers. Structural changes in pectin have been documented during orange-juice cloud destabilization, while high-pressure homogenization can markedly improve turbidity and sedimentation stability through particle-size reduction and pectin restructuring despite incomplete PME inactivation [20,21]. A complementary orange–kiwifruit study combined high-pressure processing with a natural pectin methylesterase inhibitor (PMEI) and linked PME/PMEI activity, pectin characteristics, particle size, rheology, and cloud stability during 28 d of refrigerated storage [22]. These observations establish an important interpretive limit: improved cloud stability can be consistent with PME control, but it cannot be attributed to PME inactivation alone when the process also modifies the physical organization of the matrix.

### 3.2. Polyphenol Oxidase

Polyphenol oxidase (PPO) catalyzes the oxygen-dependent oxidation of phenolic substrates to reactive quinones, which can undergo subsequent reactions leading to brown pigments and to transformation or loss of phenolic compounds. In juice matrices, the magnitude of enzymatic browning depends not only on PPO activity but also on phenolic-substrate composition and accessibility, oxygen availability, pH, and the degree of tissue or cellular disruption. Direct evidence from sugarcane juice demonstrates a substantial contribution of enzymatic oxidation to color development, while the broader fruit and vegetable literature identifies PPO–phenolic interactions as a major determinant of product quality [2,23].

Color change is nevertheless not a PPO-specific endpoint. POD activity, non-enzymatic oxidation, pigment degradation, thermal reactions, and changes in particle dispersion or light scattering can all modify instrumental color independently of PPO. A robust assessment of PPO control should therefore combine enzyme activity with a directly relevant browning or color response and, where informative, phenolic retention. Formulation is an additional source of confounding because acidulants and reducing antioxidants can suppress enzymatic browning by altering reaction conditions and substrate oxidation [24]. In sugarcane juice, ohmic-heating studies have linked PPO inactivation with color preservation and storage behavior under defined processing conditions [25]. When such formulation or co-treatment effects are present, changes in PPO activity or color describe the response of the combined process rather than an isolated effect of a single physical mechanism.

### 3.3. Peroxidase

Peroxidase (POD) catalyzes peroxide-dependent oxidation of electron-donor substrates and can contribute to oxidative deterioration, pigment modification, and color changes in fruit juices. Its technological role partially overlaps with that of PPO, but the reaction requirements are different: POD depends on peroxide availability, whereas PPO directly uses molecular oxygen for phenolic oxidation. The two enzymes therefore cannot be treated as interchangeable oxidative markers, and an identical percentage reduction in PPO and POD activity does not imply an equivalent product-level effect [2,3].

POD activity can also comprise comparatively heat-resistant fractions, further distinguishing it as a processing target. Orange peroxidase has shown substantial thermal stability, making persistence of residual activity after mild processing plausible [26]. The relevant technological question is not whether POD activity decreases, but whether the reduction is accompanied by improved pigment, color, or oxidative stability without an excessive quality penalty. In sugarcane juice, ohmic-heating studies have shown that POD inactivation, phenolic degradation, and color change vary together with processing conditions and thermal exposure [27]. A related study examining moderate electric-field effects on POD activity, phenolic compounds, and color reinforces the same point: the enzyme response must be interpreted together with the product response and with the accompanying physical or thermal process state [28]. These comparisons do not imply mechanistic equivalence with HC; they establish the type of product-level evidence needed before a POD response can be assigned technological significance.

### 3.4. Enzyme–Property Linkage and Parallel Pathways

PME, PPO, and POD therefore represent different technological targets rather than a single category of juice enzymes. PME is most directly linked to pectin functionality and cloud stability; PPO to phenolic oxidation and browning; and POD to peroxide-dependent oxidative reactions, pigment behavior, and color stability. Divergent enzyme responses have also been reported in orange juice subjected to combined ultrasound and high-pressure processing, where PME activity decreased while PPO and POD activities increased [29]. Cross-enzyme comparison based only on percentage inactivation would therefore obscure these functional differences.

Enzyme activity is therefore an intermediate response rather than a final technological endpoint. Interpretation is strongest when the enzyme response is considered together with the property it is expected to influence. Vitamin C, antioxidant capacity, sensory attributes, and microbial quality remain important for defining overall quality retention or collateral burden, but they do not by themselves demonstrate that PME, PPO, or POD control produced the observed benefit. Conversely, a favorable physicochemical response does not establish enzyme-mediated causality when the process can alter the same property through independent physical pathways.

Intensive fluid-mechanical treatments make this distinction especially important. In orange juice, pressure-driven homogenization improved stabilization despite substantial residual PME, consistent with direct changes in particle dispersion and pectin structure [21,30]. Enzyme inactivation and matrix restructuring can therefore converge on the same macroscopic endpoint. Similarly, color or phenolic retention can reflect enzyme control together with changes in substrate accessibility, oxygen or peroxide exposure, and thermal reactions. Concurrent enzyme reduction and improvement in product quality thus support a treatment-level association, not a single causal pathway.

## 4. Evidence from Hydrodynamically Cavitated Juices

Hydrodynamic cavitation does not produce a uniform PME, PPO, or POD response across real juice matrices. The magnitude and technological meaning of the enzyme response vary with matrix composition, reactor configuration, treatment duration, thermal history, and, in combined processes, formulation and temperature management. Because enzyme responses cannot be interpreted independently of the process state in which they were generated, the reactor configuration, scale, operating conditions, and thermal or formulation context of the eligible studies are summarized in Table 1. Accordingly, treatments are distinguished as HC, thermally assisted HC, or combined/hurdle-assisted processing according to the reported thermal and formulation context.

### 4.1. PME Response

Under moderate-temperature orange-juice processing, PME control remained partial. With bulk temperature maintained at approximately 33 °C, the maximum reported PME reduction was 30.38 ± 3.98%, whereas the two conditions selected by multiresponse optimization produced smaller reductions of 11.27% and 22.76%. Vitamin C retention at those optimized conditions was 94.43% and 85.71%, respectively, and antioxidant retention exceeded 93% [31]. Viscosity and color also changed during treatment, but these responses occurred together with recirculation and homogenization and therefore cannot be assigned to PME reduction alone. The result defines a quality-retention region with only partial PME control rather than complete enzyme stabilization.

A substantially stronger PME response was obtained when HC was deliberately combined with heat. At 4 bar and imposed bulk temperatures of 40–70 °C, PME inactivation increased with temperature and exposure time, reaching 65.00 ± 0.44% at 70 °C for 8 min, compared with 90.30 ± 0.09% after conventional pasteurization. At the same HC condition, the surface-weighted mean particle diameter decreased from 98.7 to 49.9 μm and no visible separation was reported during refrigerated storage. The gain in enzyme control was accompanied by a marked vitamin C loss: vitamin C decreased from 49.78 mg/100 mL in untreated juice to 16.01 mg/100 mL, close to the 15.16 mg/100 mL measured after pasteurization [32]. The 65% PME reduction therefore characterizes a thermally assisted HC treatment and cannot be interpreted as a cavitation-specific effect independent of the imposed temperature.

Tomato juice provides a contrasting example in which pronounced physical stabilization occurred while PME control remained weak. PME activity changed only modestly across the HC design and at the optimized 10 psi/10 min condition, whereas thermal processing produced 92.2% PME inactivation; bulk temperature reached approximately 47 °C at the upper end of the pressure–time design. Particle size decreased markedly and sedimentation behavior improved, while selected HC conditions retained lycopene, vitamin C, and phenolic compounds better than thermal processing [33]. The persistence of most PME activity alongside improved physical stability supports a parallel contribution from particle-size reduction and matrix homogenization rather than an interpretation based on PME inactivation alone.

A longer recirculating orange-juice treatment produced the same broad separation between enzyme response, colloidal stabilization, and quality burden. The maximum observed PME reduction was 36.36% at 4 bar for 60 min, when bulk temperature was approximately 40 °C; at 90 min and approximately 42 °C, PME reduction was slightly lower at 34.67%. Particle size decreased rapidly, from approximately 17.1 μm in untreated juice to 7.31 μm after 10 min, and physical stability during refrigerated storage improved with treatment. At the same time, vitamin C decreased from 33.83 ± 0.15 mg/100 mL in untreated juice to 16.65 ± 0.32 mg/100 mL at 60 min and 15.57 ± 0.13 mg/100 mL at 90 min [34]. Extending treatment therefore strengthened physical stabilization but progressively reduced nutritional retention without producing near-complete PME inactivation.

Huyou juice provides a particularly informative PME–pectin–turbidity linkage. At 0.5 MPa, the 40 min HC condition (HC-40) left 48.88% residual PME activity, while cloud value increased from 37.08% to 58.32%. The same treatment altered endogenous pectin molecular weight, degree of esterification, structure, rheology, and turbidity behavior [35]. PME response, pectin modification, and turbidity behavior were therefore documented within the same experimental system. However, improved turbidity stability cannot be attributed to PME reduction alone because pectin characteristics also changed during treatment and the post-treatment thermal history was not reported.

A subsequent Huyou study extended the response to all three target enzymes under comparatively low reported bulk temperature. Juice entered the process at 4 °C and post-HC temperature remained below 25 °C; PME activity decreased progressively with treatment time, while cloud value and turbidity increased and particle size and viscosity decreased [36]. Ascorbic acid and antioxidant capacity declined with longer treatment, whereas total phenolics increased and sensory and flavor attributes were generally better preserved than after thermal pasteurization. These results show that PME reduction was reported under low bulk-temperature conditions in this matrix, but the absence of a matched non-cavitating hydraulic control prevents quantitative separation of cavitation from recirculation, shear, and other hydrodynamic contributions.

### 4.2. PPO Response

PPO control was limited in several juices processed without an added formulation hurdle. In aonla juice, PPO inactivation remained modest across the HC design and at the optimized 10 psi/15 min condition; bulk temperature increased from approximately 22 to 46 °C across the design. Sedimentation, cloud behavior, particle size, and viscosity changed substantially, while the decrease in browning index was comparatively small. Selected HC conditions retained vitamin C, phenolic compounds, antioxidant activity, and sensory quality better than the 95 °C/3 min thermal comparator [37]. The process therefore improved several physical and quality attributes while leaving most PPO activity intact, which argues against assigning those changes primarily to PPO inactivation.

Much larger PPO reductions were obtained in sugarcane juice processed as a combined hurdle treatment. With ascorbic acid added before processing, precooling to 4 °C, and active temperature management, the experimentally optimized 30 min treatment produced 75.12 ± 1.40% PPO inactivation; the largest directly observed PPO inactivation in the factorial design was 86.99%. At the optimized condition, color change remained small (ΔE 2.31 ± 0.20), viscosity was 3.68 ± 0.12 cP, and total phenolics, flavonoids, and antioxidant activity were comparatively well preserved [38]. Ascorbic acid alone reduced PPO activity by approximately 6%, and precooling, continued cooling, and HC were applied together. The response therefore demonstrates strong PPO control by the combined process package rather than by cavitation in isolation.

Apple juice and fresh sugarcane juice again show that marked physical changes can occur with limited PPO inactivation. In apple juice, PPO inactivation reached approximately 13% at 103.42 kPa for 30 min, while settling decreased from 39% in untreated juice to 7% after HC and particle size fell from 8934.09 to 1112 nm. Bulk temperature increased from approximately 22 to 45 °C, and selected HC conditions retained antioxidant activity better than the thermal comparator [39]. In fresh sugarcane juice, the largest directly observed PPO inactivation in the factorial design was 18.69% at 15 psi/30 min. Numerical optimization predicted 12.69 psi/9.77 min, and experimental validation at 12.5 psi/10 min yielded 9.52% PPO inactivation [40]. In a separately reported comparison at the same nominal 12.5 psi/10 min condition, vitamin C and antioxidant capacity were 6.35 mg AAE/mL and 34.93 mg GAE/100 mL, respectively, compared with 6.77 mg AAE/mL and 36.45 mg GAE/100 mL in untreated juice [40]. Greater treatment severity progressively reduced bioactive retention. In both matrices, the technologically useful region was therefore located below the condition of maximum PPO reduction.

The later Huyou study provides an important counterpoint to the generally weak PPO response observed under milder conditions in other matrices. PPO activity decreased progressively with treatment duration and was significantly lower under the 40 and 50 min HC conditions (HC-40 and HC-50, respectively) than in both untreated and thermally pasteurized samples, while post-HC bulk temperature remained below 25 °C [36]. This result does not establish a universal low-temperature HC effect on PPO; rather, it reinforces the matrix- and reactor-specific character of the response and the need to separate bulk-temperature conditions from mechanism-specific attribution.

### 4.3. POD Response

The available POD evidence spans the same contrast between limited control under moderate conditions and stronger responses in more complex or more severe treatments. In temperature-controlled orange juice, maximum reported POD reduction was 22.22 ± 3.57%, while the two multiresponse-optimized conditions produced reductions of 11.91% and 13.79%; thermal processing at 90 °C for 30 s produced approximately 80% inactivation [31]. The mild HC conditions retained vitamin C and antioxidant capacity more effectively than the thermal comparator but left substantial POD activity, illustrating the trade-off between oxidative-enzyme control and quality preservation.

In the ascorbic-acid-assisted sugarcane process, optimized POD inactivation reached 65.21 ± 0.14% at 4 °C/30 min and the largest directly observed POD inactivation in the factorial design was 78.19% [38]. As with PPO, these values describe the combined effect of formulation, temperature management, and HC; ascorbic acid alone reduced POD activity by approximately 2.5%. In Huyou juice, POD activity also decreased progressively with HC duration at post-treatment temperatures below 25 °C, with the longer treatments significantly lower than untreated and thermally pasteurized samples [36]. Together, these results show that substantial POD reduction is possible under different treatment packages, but they do not support a single cavitation-specific mechanism or a transferable degree of inactivation.

Mixed-fruit juice provides an integrated POD example because enzyme response, thermal history, quality retention, and storage were all examined. POD inactivation increased with pressure and treatment time to 47.96 ± 0.14% at 6 bar for 60 min. Numerical optimization predicted 5.053 bar/51.857 min, and experimental validation at 5 bar/52 min yielded 40.23% POD inactivation; thermal treatment at 90 °C for 30 s produced 98.84 ± 0.53% inactivation. The highest-severity HC condition reached a bulk temperature of approximately 51.1 °C and therefore cannot be considered a low-temperature treatment [41]. At the experimentally validated 5 bar/52 min condition, vitamin C, total phenolic content, and DPPH antioxidant activity were 0.378 mg/mL, 32.22 mg GAE/100 mL, and 26.63% inhibition, respectively [41]. The selected operating condition therefore represents a compromise between partial POD control and retention of multiple quality attributes rather than a maximum-inactivation endpoint. This case provides a concrete illustration of the interpretive sequence in Figure 1. A defined HC condition produced a measurable POD response together with quality-retention outcomes, while increasing treatment severity also increased the thermal contribution. The resulting technological inference is therefore based on the balance between enzyme control and product quality under the complete processing condition, rather than on POD inactivation or cavitation intensity considered in isolation.

### 4.4. Evidence During Storage

Storage measurements reveal a distinction that is not apparent from immediate post-process results. Several studies followed appearance, sedimentation, microbial quality, or bioactive compounds during refrigeration without repeatedly measuring the target enzyme. Thermally assisted orange juice remained physically stable during refrigerated observation, and a later orange-juice treatment was followed for sedimentation over 7 d [32,34]. Huyou juice was followed for turbidity, appearance, and broader quality attributes over 14 d [35,36]. These observations can demonstrate persistence of physical or overall product quality, but they do not establish persistence of PME, PPO, or POD control when enzyme activity itself is not remeasured.

Where enzyme activity was reassessed during storage, the apparent degree of control could weaken. Residual PME activity increased slightly in tomato juice during refrigerated storage [33]. PPO inactivation also declined during refrigerated storage in apple and fresh sugarcane juices [39,40]. These observations show that persistence of enzyme control is a distinct outcome from the immediate post-process response.

Enzyme control, product stability, and shelf-life performance should therefore be treated as linked but distinct outcomes.

The experimental controls available across the eligible studies determine the level at which these responses can be interpreted. Untreated samples and conventional thermal treatments permit treatment-level comparisons but do not isolate cavitation from concurrent hydraulic, thermal, and formulation effects. Evidence strength was therefore appraised according to the causal resolution supported by the available control structure rather than by an aggregate study-quality score. The control structure and the resulting attribution boundaries for the available evidence are summarized in Table 2.

## 5. Within-Study Quantitative Comparisons

Because PME, PPO, and POD responses were obtained in different juice matrices, assays, reactor configurations, and processing conditions, quantitative comparison was restricted to within-study, same-enzyme contrasts. For each comparison, the experimentally measured response at a selected or experimentally validated condition was compared with the largest directly observed inactivation of the same enzyme within the same study. This approach avoids quantitative comparison across non-equivalent enzymes, matrices, assays, and processing systems. The resulting contrasts are summarized in Table 3.

Four of the 11 primary studies provided the information required for this exact within-study comparison, yielding six enzyme-level contrasts. In all six contrasts, inactivation at the selected or experimentally validated condition was numerically lower than the largest directly observed value for the same enzyme within the same study. Because two studies contributed two enzyme contrasts each, the six contrasts do not represent six independent study-level replications; the 6/6 pattern is therefore descriptive. Within these directly comparable studies, the selected or experimentally validated condition did not coincide with the condition yielding the largest directly observed enzyme inactivation.

Separately, two publications provided three same-condition PPO trajectories with exact measurements at day 0 and at the end of refrigerated storage [39,40]. End-of-storage inactivation was numerically lower than the corresponding day-0 value in all three trajectories. Two trajectories originated from the same sugarcane study and therefore do not represent independent study-level replications; the 3/3 pattern is descriptive, and no pooled storage estimate was calculated.

## 6. Enzyme Control–Quality Retention Trade-Off

The within-study quantitative pattern identified above becomes technologically meaningful only when interpreted against product function and quality burden. The physicochemical endpoints most relevant to PME, PPO, and POD function and reported within the same studies are summarized in Table 4. Linkage was graded as relatively direct when the endpoint reflected the enzyme substrate or an immediate functional consequence, functionally relevant but non-specific when the endpoint was technologically linked to enzyme action but could arise through parallel pathways, and indirect when multiple independent mechanisms could readily produce the same response.

### 6.1. Incomplete Enzyme Control

Substantial residual enzyme activity was reported under several mild or temperature-limited HC conditions. Moderate-temperature orange juice, tomato juice, and aonla juice retained most of the measured PME, POD, or PPO activity even though physical or quality attributes changed measurably [31,33,37]. Apple juice, longer recirculating orange processing, and fresh sugarcane juice show the same broad separation between limited enzyme control and pronounced changes in settling, particle size, rheology, or related product attributes [34,39,40]. These results do not indicate that mild HC is without value; they show that a useful product response can occur without extensive enzyme inactivation.

This distinction is most evident for PME-related physical stability. Particle-size reduction, pectin restructuring, and altered interactions within the dispersed phase can improve cloud or sedimentation behavior while substantial PME activity remains. Pressure-driven homogenization in orange juice provides independent evidence that cloud stabilization can arise through direct matrix modification without near-complete PME inactivation [21,30]. The Huyou results extend this interpretation within HC because PME reduction, pectin modification, and improved turbidity behavior occurred in the same matrix, while the treatment also changed pectin structure [35]. Physical stabilization can therefore be consistent with PME control without establishing that the stabilization was mediated primarily by PME inactivation.

The same principle applies to PPO and POD. Color and oxidative stability depend not only on residual enzyme activity but also on substrate availability, oxygen or peroxide exposure, formulation, temperature, and matrix structure. A condition that leaves considerable PPO or POD activity may still preserve acceptable product quality over a limited period, whereas the same residual activity may be insufficient when the objective is durable suppression of enzymatic deterioration. The technological meaning of incomplete enzyme control is therefore function-specific and cannot be inferred from the inactivation percentage in isolation.

### 6.2. Thermally Assisted and Hurdle-Assisted Enzyme Control

Stronger enzyme control was observed under thermally assisted, higher-severity, or hurdle-assisted conditions in several matrices. Thermally assisted orange processing produced substantially greater PME reduction than the lower-temperature orange treatments; the ascorbic-acid-assisted, actively cooled sugarcane process produced large PPO and POD reductions; and the mixed-fruit system reached its greatest POD reduction after prolonged treatment accompanied by substantial bulk heating [32,38,41]. These cases demonstrate that stronger control is attainable, but the relevant unit of interpretation is the complete treatment package rather than cavitation considered in isolation.

Temperature is particularly important because it can affect both enzyme-inactivation kinetics and the cavitating flow itself [8]. Juice-processing studies outside HC likewise show that pressure, temperature, enzyme fraction, and treatment combinations can jointly determine PME inactivation and its persistence [9,10,42].

Combined-hurdle processing imposes the same interpretive limit. In sugarcane juice, ascorbic acid, precooling, active cooling, and HC were applied together, and ascorbic acid itself altered PPO and POD activity [38]. The appropriate conclusion is therefore that the combined process achieved strong enzyme control. Conversely, Huyou juice showed progressive changes in PME, PPO, and POD while reported post-HC bulk temperature remained below 25 °C [36]. This demonstrates that measurable enzyme reduction was reported under low bulk-temperature conditions in this matrix; it does not exclude local thermal contributions or establish a cavitation-specific mechanism.

### 6.3. Quality Burden with Increasing Severity

In several studies, increasing treatment severity was associated with greater enzyme control, but the quality response was multidimensional and did not necessarily move in the same direction. Longer treatment, higher imposed temperature, or greater recirculation exposure can increase bulk heating and losses of heat- or oxidation-sensitive compounds, while other attributes such as colloidal stability, phenolic extractability, sensory properties, or microbial quality may remain stable or improve. A processing condition should therefore be judged against the quality attributes that define the intended product rather than against a single generic measure of quality retention.

The optimization evidence therefore indicates a severity–quality trade-off rather than a simple failure to maximize enzyme inactivation. Once physical properties, retention of bioactive compounds, color, microbial quality, or sensory response enter the selection criterion, additional enzyme reduction is technologically useful only if the resulting product continues to meet the quality requirements of the intended application.

The same enzyme–quality trade-off is evident in other non-thermal juice-processing technologies. High-pressure processing can achieve substantial endogenous-enzyme control while retaining physicochemical and nutritional quality, although the response depends on pressure, treatment time, temperature, and enzyme-specific resistance [9,10,43]. Pulsed electric field processing can likewise reduce endogenous enzyme activity while limiting changes in physicochemical, nutritional, and sensory attributes under appropriately selected conditions; its outcome depends on process variables including electric-field strength, pulse characteristics, treatment time, and specific energy [44]. These comparisons do not imply mechanistic equivalence with HC. They show that, across different processing technologies, enzyme control must be evaluated together with process exposure and the associated product-quality response.

Thermally assisted orange juice illustrates this trade-off directly: stronger PME control at higher imposed temperature was accompanied by progressively greater vitamin C loss [32]. Longer recirculating orange processing and higher-severity sugarcane treatment likewise reduced retention of vitamin C or other bioactive compounds as exposure increased [34,40]. The Huyou studies further illustrate that product responses need not change uniformly. Improved colloidal stability was documented in the PME–pectin-focused study, whereas the subsequent multi-enzyme study reported declining ascorbic acid and antioxidant capacity alongside increases in total phenolics and comparatively favorable sensory and flavor attributes [35,36]. No single quality response can therefore define the technological optimum. The principal quality-retention responses reported across increasing, selected, or reference treatment conditions are summarized in Table 5. Only experimentally measured quality outcomes are tabulated; model-predicted optimum values are not presented as measured responses.

Condition-level quantitative pairing is available within individual studies, but it does not define a transferable enzyme–quality relationship. In orange juice, two multiresponse-selected conditions produced PME reductions of 11.27% and 22.76%, while vitamin C retention was 94.43% and 85.71%, respectively; POD reductions were 11.91% and 13.79%, with antioxidant retention of 95.05% and 93.38% [31]. This within-study contrast is consistent with an enzyme-control–quality-retention trade-off, but it does not establish that the quality change was caused by enzyme inactivation because treatment conditions changed simultaneously. Across studies, PME, PPO, and POD were measured using different assays and reporting scales, while quality was represented by non-equivalent endpoints including nutrient retention, antioxidant capacity, physical stability, color, and sensory acceptability under different reactor, thermal, and formulation conditions. These data do not provide a common biologically interpretable effect measure for a defensible pooled meta-regression or transferable mathematical model. Quantitative enzyme–quality models require paired condition-level measurements within a defined matrix–enzyme–reactor system using consistent enzyme assays, predefined quality endpoints, controlled process variables, and reported uncertainty. 

Storage adds a further constraint because an operating condition that is favorable immediately after processing may not maintain the same enzyme response over time. Exact same-condition PPO measurements were available for apple and fresh sugarcane juices [39,40]. The three exact storage trajectories are summarized in Table 6.

### 6.4. Matrix-, Enzyme-, and Reactor-Specific Operating Windows

The available evidence does not support a universal HC pressure, treatment time, cavitation number, or temperature for enzyme control in juices. A useful operating window is instead the range of conditions in which the target enzyme is controlled sufficiently for a defined product function, the directly relevant physicochemical property is favorable, the required quality attributes are retained, and adequate enzyme control together with the associated product function is maintained over the intended storage period.

Cross-matrix interpretation requires characterization of the juice before HC treatment. Source-reported baseline descriptors for acidity, soluble solids or formulation, physical matrix structure, and initial enzyme status are summarized in Table 7.

These descriptors do not permit quantitative isolation of independent matrix effects across studies. Matrix identity varies together with target enzyme, assay definition, reactor configuration, hydraulic exposure, and thermal history. Cross-study regression would therefore confound matrix effects with process and analytical differences.

Matrix and enzyme specificity are intrinsic to this problem. PME acts through pectin and dispersed-phase organization; PPO depends on phenolic substrates and oxygen availability; POD depends on peroxide availability and exhibits distinct resistance characteristics. Reported percentage reductions across these enzymes and juices therefore cannot be treated as values on a common response scale. A degree of PME reduction associated with a favorable cloud-stability response in one citrus matrix has no direct quantitative equivalence to a PPO or POD reduction associated with color or oxidative stability in another juice.

Reactor specificity further limits transferability because cavitation inception and development depend on reactor geometry and operating state, while temperature can further modify cavitation behavior [4,6,8]. Conditions identified in one reactor should therefore not be transferred as general HC settings without re-establishing the relevant hydraulic and thermal state. Several eligible publications originate from related research groups and reactor architectures; publication count should therefore not be interpreted as an equivalent number of independent cross-platform replications. Independent validation across reactor architectures remains limited.

Huyou juice illustrates both the value and the present limit of an enzyme-specific operating-window approach. PME activity, pectin characteristics, and turbidity behavior were measured within the same matrix, providing an integrated same-study assessment of enzyme response and product structure [35]. The later Huyou work extended the same matrix to simultaneous PME, PPO, and POD responses under comparatively low reported bulk temperature [36]. Even in this comparatively integrated evidence, concurrent matrix restructuring and incomplete separation of the hydraulic mechanisms prevent the response from being reduced to a single enzyme pathway or a cavitation-specific cause.

The practical implication is not to search for a universal HC setting, but to define the minimum enzyme control required for a specific product function and then identify the least burdensome processing conditions that achieve it reproducibly. Such windows must be established for the relevant matrix, enzyme, and reactor and verified against the associated physicochemical response, quality burden, and storage persistence.

## 7. Evidence Needed to Define Reliable Operating Windows

The evidence summarized above identifies a consistent methodological gap: enzyme activity, the process state that produced it, the directly relevant product property, quality burden, and storage persistence are rarely characterized with equal resolution in the same experiment. Reliable operating windows require these elements to be connected rather than optimized separately. The level of experimental detail must also match the strength of the intended conclusion: demonstrating treatment performance requires less causal resolution than attributing the response specifically to cavitation or transferring a condition to another matrix or reactor.

### 7.1. Process Characterization

A reproducible process description includes reactor type and constriction geometry, the relevant upstream and downstream pressures, pressure drop, flow rate, treated volume, pump–loop configuration, the variable that best represents cumulative exposure, such as treatment duration, number of passes, recirculation history, or residence-related exposure, and specific energy input when reported. 

Cavitation number, denoted here as $C_{v}$, is commonly expressed in dimensionless form as

(1) $$C_{v}=\frac{P_{ref}-P_{v}}{\frac{1}{2}\rho U_{ref}^{2}}$$

where $P_{ref}$ is the pressure used as the reference state, $P_{v}$ is the liquid vapor pressure, ρ is the liquid density, and $U_{ref}$ is the characteristic flow velocity [4,5]. Published HC studies do not use completely uniform pressure and velocity definitions, so the calculated value depends on the selected reference pressure, characteristic velocity, and temperature-dependent vapor pressure [4,5]. Reactor geometry is also not represented explicitly in the conventional expression, although it strongly affects cavitation inception and extent [5,6]. A recent parametric Venturi study further demonstrated numerically that throat geometry can be treated as an adjustable design variable for identifying a configuration associated with cavitation inception under defined hydraulic conditions [45]. Cavitation number should therefore be reported together with its calculation basis and reactor and operating details rather than used as a universal severity descriptor [4,5,6].

Numerical and CFD studies provide complementary information on reactor-specific hydrodynamics that is not captured by a single cavitation number [5,6,7]. Cavitation inception and spatial extent are sensitive to reactor geometry, while CFD can resolve local hydrodynamic quantities such as shear stress that coexist with cavitation [6,7]. These studies support the use of modeling to characterize reactor-specific flow conditions and to interpret the limited transferability of cavitation number across different configurations [5,6,7]. However, simulation-derived hydrodynamic fields do not by themselves establish enzyme-specific effects and should be linked to experimental measurements obtained under the corresponding processing conditions. Experimental flow diagnostics can complement these simulations; synchrotron X-ray particle image velocimetry has been applied to cavitating Venturi flow to obtain simultaneous velocity and void-fraction fields that are not represented by cavitation number alone [46].

Experimental evidence from a Venturi-type reactor further showed that cavitation performance, including intensity and unsteady behavior, is jointly influenced by cavitation number, Reynolds number, and a thermodynamic parameter accounting for thermal effects, while cavitation length increased and then decreased with increasing temperature [47].

Thermal characterization is equally important. Initial, maximum, and final bulk temperatures are most informative when accompanied by the time–temperature trajectory whenever appreciable heating occurs or temperature is intentionally controlled. External heating, cooling, precooling, jackets, baths, and interruptions in circulation form part of that thermal history. Temperature affects enzyme-inactivation kinetics and can also alter cavitation intensity and regime [7,8]. A single final temperature cannot distinguish a short transient peak from prolonged exposure and is therefore inadequate for separating temperature-limited HC from thermally assisted processing.

Accordingly, cross-device standardization should use a multidimensional process-state descriptor comprising reactor geometry, upstream and downstream pressures and pressure drop, flow rate, complete time–temperature history, cumulative exposure, and the calculation basis for $C_{v}$, rather than an unvalidated scalar severity index [4,5,6]. A composite index should only be introduced after its weighting and transferability have been validated against common experimental endpoints across different reactors and matrices.

### 7.2. Mechanistic Decoupling of Cavitation, Thermal, and Fluid-Mechanical Contributions

Mechanistic decoupling should be treated as an experimental-design problem rather than inferred from differences among nominal operating conditions. An untreated sample establishes the response of the complete HC-based treatment relative to the unprocessed matrix. A conventional thermal comparator permits process-level comparison with heat-based stabilization, particularly for enzyme reduction and quality retention. Neither comparison isolates cavitation from pumping, recirculation, shear, homogenization, or the thermal history generated during HC. Differences between HC and pasteurization should therefore be interpreted as process-level contrasts rather than as quantitative estimates of a cavitation-specific contribution.

Mechanism-specific attribution is strengthened by combining the full HC condition with a non-cavitating hydraulic comparator and, where thermal contributions are relevant, a separate thermal-history-matched comparator. The hydraulic comparator should preserve the same pump–loop configuration, treated volume, cumulative flow or residence exposure, pump work, and bulk thermal history as closely as practicable while operating below cavitation inception. The thermal comparator should reproduce the measured bulk time–temperature trajectory without the cavitating hydraulic exposure. These controls can help distinguish the response of the complete treatment from responses associated predominantly with thermal or hydraulic conditions, but they cannot create a perfectly identical state differing only in the presence or absence of cavitation because suppression of cavitation necessarily modifies part of the pressure and flow field.

For orifice- and Venturi-type systems, one practical approach is to adjust upstream or downstream pressure conditions sufficiently to move the operating point below cavitation inception while matching flow rate and cumulative hydraulic exposure as closely as practicable [5,6]. Pressure drop, flow rate, pump work, recirculation history, and temperature trajectory should therefore be measured for both cavitating and non-cavitating conditions. Hydrodynamic stresses coexist with cavitating flow and can contribute independently to the observed treatment response [7]. A non-cavitating control should consequently be interpreted as a closely matched hydraulic comparator rather than as an otherwise identical replica of the cavitating state.

Parameter isolation should also account for interactions among operating conditions and deliberately introduced hurdles. When formulation, precooling, active cooling, or another treatment component is incorporated, the measured response belongs to the complete treatment package unless the contribution of each component is resolved experimentally. The large PPO and POD reductions reported for ascorbic-acid-treated, temperature-managed sugarcane juice illustrate this attribution boundary [38]. Factorial or staged experimental designs can test the contributions and interactions of pressure conditions, treatment duration, thermal management, and formulation, provided that the same enzyme and product endpoints are measured across the relevant conditions.

Mathematical modeling provides a complementary layer of evidence rather than a substitute for experimental decoupling. CFD can characterize reactor-specific pressure and velocity fields, cavitation inception and spatial extent, and coexisting shear fields that are not represented by a single cavitation number [5,6,7]. Enzyme-kinetic models can separately describe the dependence of enzyme response on defined pressure, temperature, and time exposures [9,32]. These approaches can identify differences in process state and guide the selection of informative experimental comparisons, but model fit alone cannot establish the causal contribution of cavitation. Mechanism-specific inference therefore requires consistency among process characterization, appropriately matched controls, and experimentally measured enzyme and product responses.

### 7.3. Enzyme–Function Linkage, Optimization, and Experimental Validation

A meaningful optimization begins with a defined product function. For PME, the most informative endpoints are pectin characteristics together with cloud stability, turbidity, sedimentation, or rheology. For PPO, enzyme activity is most informative when interpreted with browning, color, and phenolic oxidation; for POD, the corresponding evidence concerns pigment, color, and oxidative stability. These measurements determine whether the enzyme response is accompanied by the product response for which the enzyme is technologically relevant.

Analytical reporting is most informative when it preserves the original enzyme information: assay conditions, activity units, untreated baseline, residual activity or inactivation calculation, and statistical uncertainty. Percentage inactivation is useful within a study but is not a common scale across enzymes, matrices, or assay methods. Experimentally measured values, validated operating conditions, model-derived predictions, and recalculated quantities need to remain distinguishable, with condition-level numerical results reported directly whenever they support quantitative conclusions.

Quality measurements characterize the product-level burden associated with the processing condition. Vitamin C, phenolic content, antioxidant capacity, sensory attributes, microbial quality, and related endpoints can identify preservation or collateral damage, but they are not substitutes for the physicochemical property directly relevant to the target enzyme. The relevant question is whether the directly relevant product function is favorable while the attributes that define the intended product remain within an acceptable range.

Microbial control should be treated as a parallel process-performance criterion rather than as a surrogate for enzyme control. Juice studies measuring both domains within the same processing system reported microbial reduction together with endogenous-enzyme inactivation and product-quality responses [36,39,41]. These outcomes represent distinct performance criteria and should therefore be evaluated separately. Adequate enzyme control does not establish microbiological adequacy, and microbial reduction does not establish control of PME, PPO, or POD. A technologically useful operating window should therefore evaluate microbial performance alongside enzyme-linked physicochemical function and quality retention using endpoints and acceptance criteria appropriate to the intended juice product.

Multiresponse optimization can then be used to locate a practical operating region, but the experimentally selected condition and the condition of maximum enzyme reduction must remain separate quantities. The direct within-study contrasts show why optimization should include the physicochemical property directly relevant to the target enzyme and the relevant quality burden rather than maximizing enzyme inactivation in isolation.

A model-derived optimum requires experimental testing under the predicted conditions, with the measured validation result kept separate from the model estimate. A well-fitted pressure–time surface can identify a useful treatment condition without resolving the contributions of temperature, recirculation, formulation, or cavitation itself. Optimization identifies where to operate; experimental validation establishes whether the predicted response is reproduced, whereas appropriately matched controls determine the level of causal attribution that is justified.

### 7.4. Storage Persistence

Any claim that enzyme control contributes to stability during storage requires evidence beyond the immediate post-process measurement. Repeated measurements of enzyme activity should be paired with the physicochemical property expected to be influenced by that enzyme over the intended storage period under defined packaging and storage conditions. Broader shelf-life claims additionally require the microbiological, sensory, and compositional endpoints that define product acceptability. This requirement follows directly from the increase in residual PME activity in tomato juice and the weaker PPO control observed during storage in apple and fresh sugarcane juices [33,39,40].

In the mixed-fruit study, experimental validation of the 5 bar/52 min condition yielded 40.23% POD inactivation, whereas 31.87% was reported for HC-treated juice at the same nominal condition after 14 days of refrigerated storage; sedimentation and color difference increased and bioactive compounds declined during storage [41].

The sampling schedule should distinguish a stable response from progressive changes in residual activity, delayed deterioration, or analytical variability. Activation of latent enzyme forms associated with conformational changes has been reported for thermally treated apple-juice PPO [48]. Recovery of PPO and POD activity during storage after pulsed-light processing has also been interpreted as compatible with reversible conformational changes [49]. These observations derive from other juice-processing technologies and do not demonstrate the mechanism responsible for apparent activity recovery in HC-treated juices. Apparent recovery should therefore not be interpreted automatically as true molecular reactivation. Enzyme and product trajectories should be interpreted together because physical stability or color can change through pathways that are not enzyme-mediated.

HC studies that reassessed enzyme activity during storage were short refrigerated investigations with sparse or nonuniform sampling schedules [33,39,40]. The mixed-fruit study likewise reported a 14-day refrigerated endpoint [41]. These data do not support a transferable predictive model across storage conditions; such models require repeated paired measurements of enzyme activity and the relevant product-quality endpoints over multiple storage times and conditions, followed by independent validation.

### 7.5. Transferability Across Matrices, Reactors, and Scale

Transferability requires validation in the matrix, for the enzyme, and in the reactor for which the operating window is intended. Independent juice lots are important for establishing robustness within a matrix, whereas validation in another matrix or reactor addresses a different level of transfer. Enzyme stability, substrate availability, pectin behavior, and rheology can change with matrix composition, while the hydraulic state depends on reactor geometry and operating configuration.

Transfer to another geometry or scale therefore represents a new validation problem rather than direct application of the same nominal pressure, treatment time, or cavitation number. Reactor geometry affects cavitation inception and development [4,5,6]. Temperature can further shift cavitation behavior [8]. A transferable process description requires enough hydraulic and thermal information to reproduce the relevant process state and then demonstrate that the enzyme, physicochemical-property, and quality responses meet predefined performance criteria under the new conditions.

Scale-up introduces additional constraints because changes in reactor dimensions, flow capacity, and operating configuration can modify the pressure field and the development of cavitation [17,50]. Consequently, nominal pressure drop or cavitation number should not be assumed to represent an equivalent hydrodynamic state when a process is transferred from bench to pilot or industrial scale [6,50]. Scale-up should therefore aim to reproduce the relevant hydraulic and thermal process state rather than rely on geometric enlargement or direct transfer of nominal operating settings. Flow rate and achievable throughput, pressure conditions, temperature history, and specific energy demand should be characterized at the new scale, and the corresponding enzyme and product responses should be experimentally revalidated [13,17,51].

Real-scale feasibility has also been demonstrated in beverage processing. HC-assisted brewing was investigated in a dedicated 230 L microbrewery-scale unit, and the system design was described as allowing single-unit upscaling to the order of 10,000 L and integration of pumps and HC reactors into existing brewing plants [52].

Industrial translation additionally requires assessment of equipment durability during sustained operation, maintenance requirements and associated costs, and compatibility with the hydraulic and thermal demands of existing processing lines. These engineering criteria should be evaluated at pilot or continuous scale together with treatment performance, because short laboratory runs do not establish long-term operability or economic viability [51,53].

### 7.6. Limitations of the Available Evidence

Several limitations constrain the strength and transferability of the available evidence. The primary evidence base is small and heterogeneous in juice matrix, enzyme assay, reactor geometry, operating conditions, thermal history, formulation, and cumulative exposure. Hydraulic controls operated without cavitation and matched thermal controls are uncommon, while storage datasets are sparse and use nonuniform sampling schedules. Several publications also come from related research groups and a limited range of reactor architectures. These limitations do not support robust pooling across studies, a universal severity ranking, or specific attribution of observed effects to cavitation. Interpretation should therefore remain specific to the matrix, enzyme, reactor, and treatment conditions.

The three principal knowledge gaps, corresponding research priorities, and feasible validation methods are summarized in Table 8.

## 8. Conclusions

Hydrodynamic cavitation can modify PME, PPO, and POD activity in real juice matrices, but neither the magnitude nor the technological meaning of that response is uniform. Substantial residual activity remained after several mild or temperature-limited treatments, whereas stronger enzyme reduction was observed under thermally assisted, higher-severity, or hurdle-assisted conditions. The three enzymes must also be interpreted separately because their technological roles, substrates, resistance characteristics, and relevant product endpoints are not interchangeable.

The separation between maximum enzyme inactivation and process-selected conditions is also evident in the direct within-study comparisons: in all six enzyme-level contrasts, inactivation at the selected or experimentally validated condition was numerically lower than the largest directly observed value for the same enzyme. Physical stabilization can improve despite limited PME or PPO inactivation, consistent with concurrent changes in particle size, pectin organization, rheology, and dispersed-phase structure during HC-based processing. Conversely, stronger enzyme control can lose technological value when greater severity is accompanied by greater loss of heat- or oxidation-sensitive quality attributes. The processing objective is therefore sufficient control for the required physicochemical function with an acceptable overall quality burden, rather than maximum enzyme inactivation as an isolated endpoint.

Storage persistence and causal attribution impose two additional limits. In all three exact same-condition PPO storage trajectories, inactivation was numerically lower at the end of refrigerated storage than at day 0, reinforcing that an immediate post-process response alone does not establish durable control. At the same time, most experiments support the response of the complete HC-based treatment more strongly than the independent contribution of cavitation itself because temperature, recirculation, shear, homogenization, and formulation are not routinely isolated by matched controls. This defines the level of inference supported by the available experiments and explains why favorable results cannot be transferred directly between matrices or reactor configurations.

The scientifically useful endpoint is therefore not a universal pressure, treatment time, cavitation number, or temperature, but a matrix-, enzyme-, and reactor-specific operating window. Such a window is defined by sufficient control of the target enzyme for a specified product function, a favorable response of the directly relevant physicochemical property, acceptable retention of product-defining quality attributes, and maintenance of adequate enzyme control and product function over the intended storage period. Establishing these windows requires reproducible hydraulic and thermal characterization, controls matched to the causal claim, paired measurements of enzyme response and the directly relevant product function, experimental validation of optimized conditions, and storage verification. This framework provides a practical basis for comparing HC conditions on technological rather than merely enzymatic grounds and redirects future research from demonstrating that enzyme activity can change toward determining when that change is reproducible, durable, functionally relevant, and attributable with sufficient confidence.

## Figures and Tables

**Table 1:** Hydrodynamic cavitation processing configuration, operating conditions, and thermal/formulation context of studies reporting PME, PPO, and/or POD responses in real juice matrices.

Juice Matrix/ Target Enzyme(s)	HC Configuration and Scale	Operating Conditions	Bulk Thermal/Formulation Context
Orange (Nagpur); PME, POD [31]	Orifice; single 2 mm hole; 5 L recirculation; 1.1 kW positive-displacement pump	3–15 bar; 5–25 min	Bulk temperature maintained at ~33 °C by cold-water circulation through a double-jacketed reservoir; immediate post-treatment cooling
Orange (Valencia, Brazil); PME [32]	Orifice; 24 × 0.45 mm holes; 1.5 L system; 1.2 L recirculation tank; 1.5 CV centrifugal pump; 4 m^3^/h	4 bar; *Cv* = 0.191; 2–8 min	40–70 °C deliberately imposed and maintained by external hot-water recirculation
Tomato; PME [33]	Venturi; 5 mm throat; 1 L treated in a 2 L holding system; 0.5 HP pump	5–15 psi; *Cv* = 0.67–0.94; 5–30 min	Initial 23 ± 2 °C; maximum final temperature 47 ± 2 °C; no active temperature control described
Orange (Valencia, Brazil); PME [34]	Orifice; 16 × 1 mm holes; 1.5 L processed; 1.2 L recirculation tank; 1.5 CV centrifugal pump; 4 m^3^/h	4 bar; 10–90 min	Bulk temperature increased from ~25 to ~42 °C across treatment times despite ice-water circulation used to limit heating
Huyou turbid juice; PME [35]	Self-assembled Venturi HC system; 40 L batch	0.5 MPa; 10–50 min	Juice maintained at 4 °C before HC; post-HC time–temperature history not reported
Huyou juice; PME, PPO, POD [36]	Self-assembled HC system; 40 L sample-container capacity	0.5 MPa; 10–50 min	Initial 4 °C; post-HC bulk temperature < 25 °C
Aonla (Indian gooseberry); PPO [37]	Venturi; 5 mm throat; 2 L holding tank; 0.5 HP centrifugal pump; recirculation	5–15 psi; *Cv* = 0.69–0.94; 5–30 min	Bulk temperature increased from ~22 to ~46 °C across the factorial design; no active temperature control described
Sugarcane + 30 mg/100 mL AA; PPO, POD [38]	Orifice; 17 × 1 mm holes; 8 L holding tank; 1.5 kW centrifugal pump; recirculating system	0.35 MPa; 10–40 min	AA added before HC; juice precooled to 4, 17, or 30 °C; refrigerated jacket and PID control used for precooling and removal of treatment-generated heat
Apple (Delicious Red); PPO [39]	Venturi; 5 mm throat; 2 L; 0.5 HP reciprocating pump with VFD	34.47–103.42 kPa; 5–30 min	Initial ~22 °C; final temperature up to ~45 °C; temperature could not be actively controlled
Fresh sugarcane; PPO [40]	Venturi; 5 mm throat; 2 L sample container; 0.5 HP centrifugal pump; recirculation	5–15 psi; *Cv* decreased from 0.87 to 0.62 with increasing pressure; 5–30 min	Bulk temperature increased from ~22 to ~47 °C across the design; no active temperature control described
Mixed-fruit juice (60% mandarin, 25% pineapple, 15% watermelon); POD [41]	Orifice; 4 × 1 mm holes; 3 L system; centrifugal pump	4–6 bar; 40–60 min	Bulk temperature increased with treatment severity, reaching 51.1 °C; no active cooling described

Note: HC, hydrodynamic cavitation; PME, pectin methylesterase; PPO, polyphenol oxidase; POD, peroxidase; AA, ascorbic acid; *Cv*, cavitation number; CV, metric horsepower (cavalo-vapor); HP, horsepower; PID, proportional–integral–derivative; VFD, variable-frequency drive. Pressure units and *Cv* values are retained as reported in the original studies and should not be interpreted as directly comparable measures of treatment severity across different reactor geometries and process configurations. Temperatures refer to measured bulk-liquid temperatures and do not represent transient local conditions associated with cavity collapse. Where the source specifies equipment or container capacity rather than an unequivocal treated volume, the terms capacity or holding system are retained. The designation no active temperature control described indicates that no active heating or cooling arrangement was reported for the HC treatment and does not imply isothermal operation.

**Table 2:** Experimental control structure, evidence strength, and attribution boundaries in hydrodynamic cavitation studies reporting PME, PPO, and/or POD responses in real juice matrices.

Juice Matrix/ Target Enzyme(s)	Experimental Control/ Comparator Structure	Evidence Strength/Attribution Boundary
Orange (Nagpur); PME, POD [31]	Fresh untreated juice; thermal pasteurization at 90 °C/30 s	Permits comparison of the complete HC treatment with untreated and thermal processing. Cavitation-specific effects remain inseparable from recirculation, pumping, and associated hydraulic/mechanical exposure
Orange (Valencia, Brazil); PME [32]	Untreated juice; a control experiment without cavitation; thermal pasteurization at 90 °C/90 s	The non-cavitating control strengthens the design, but sufficiently detailed matching of hydraulic and thermal exposure is not established. Because 40–70 °C was deliberately imposed during HC, the enzyme response represents thermally assisted HC, not an HC-only effect
Tomato; PME [33]	Untreated juice; thermal comparator at 90 °C/90 s	Supports treatment-level comparison with untreated and thermally processed juice. Cavitation is not isolated from pumping/recirculation, homogenization, and concomitant bulk heating
Orange (Valencia, Brazil); PME [34]	Fresh untreated juice; commercial market juice used in selected physical comparisons; no conventional thermal comparator	Supports the response of the HC-based treatment as a whole. The commercial sample is not a process-matched control, and recirculation, homogenization, and residual bulk heating are not separated from cavitation
Huyou turbid juice; PME [35]	Original untreated juice (HC-0); no conventional thermal comparator	Supports treatment-time associations relative to untreated juice, not cavitation-specific causality. Changes in endogenous pectin provide a parallel pathway, so improved turbidity stability cannot be assigned solely to PME reduction
Huyou juice; PME, PPO, POD [36]	Untreated juice (HC-0); thermal pasteurization at 85 °C/30 s	Permits overall HC-versus-thermal process comparison. Without a matched non-cavitating hydraulic control, recirculation, shear, and other hydrodynamic contributions cannot be quantitatively separated from cavitation
Aonla (Indian gooseberry); PPO [37]	Untreated juice; thermal treatment at 95 °C/3 min	Supports overall comparison with untreated and thermal processing. Bulk heating and hydraulic homogenization/particle-size reduction accompany HC, so physical stabilization cannot be assigned specifically to PPO inactivation
Sugarcane + 30 mg/100 mL AA; PPO, POD [38]	Reference/untreated values reported within an AA-treated, temperature-managed processing design; no clean HC-only or conventional thermal comparator	AA addition, precooling, active temperature management, and HC constitute a combined treatment package. PPO and POD responses therefore cannot be quantitatively attributed to HC alone
Apple (Delicious Red); PPO [39]	Untreated juice; thermal treatment at 90 °C/2.5 min	Supports overall treatment comparison. Bulk heating and hydraulic homogenization occur concurrently, preventing isolation of cavitation-specific effects and preventing physical stabilization from being assigned specifically to PPO reduction
Fresh sugarcane; PPO [40]	Untreated juice; thermal comparator at 90 °C	Supports treatment-level comparison with untreated and thermally processed juice. Bulk heating, recirculation, and homogenization accompany HC and are not independently controlled
Mixed-fruit juice (60% mandarin, 25% pineapple, 15% watermelon); POD [41]	Untreated juice; thermal treatment at 90 °C/30 s	Supports comparison of the complete HC treatment with untreated and thermal processing. At greater HC severity, substantial bulk heating accompanies hydraulic/mechanical exposure; POD reduction is therefore a treatment-level response rather than an isolated cavitation effect

Note: HC, hydrodynamic cavitation; PME, pectin methylesterase; PPO, polyphenol oxidase; POD, peroxidase; AA, ascorbic acid. A non-cavitating control was reported, but sufficiently detailed matching of hydraulic and thermal exposure was not established [32].

**Table 3:** Exact experimentally measured within-study contrasts between selected or experimentally validated conditions and the largest directly observed enzyme inactivation.

Juice Matrix/Study	Enzyme	Selected or Experimentally Validated Condition	Largest Directly Observed Inactivation
Orange [31]	PME	5 bar/15 min: 11.27%	30.38 ± 3.98%
Orange [31]	POD	5 bar/15 min: 11.91%	22.22 ± 3.57%
AA-treated, temperature-managed sugarcane [38]	PPO	Validated 4 °C/30 min: 75.12 ± 1.40%	86.99%
AA-treated, temperature-managed sugarcane [38]	POD	Validated 4 °C/30 min: 65.21 ± 0.14%	78.19%
Fresh sugarcane [40]	PPO	Experimental validation at 12.5 psi/10 min: 9.52%	18.69%
Mixed-fruit juice [41]	POD	Experimental validation at 5 bar/52 min: 40.23%	47.96 ± 0.14%

Note: AA, ascorbic acid; PME, pectin methylesterase; PPO, polyphenol oxidase; POD, peroxidase. Only unambiguous, experimentally reported, within-study, same-enzyme values were compared; model-derived predictions were excluded. Contrasts from the same study are not independent, and no pooling across enzymes, matrices, studies, or reactor configurations was performed. The optimized condition with the highest overall desirability was used for the orange-juice study [31]. The sugarcane study represents the combined AA + temperature-management + HC treatment [38]. For study [40], numerical optimization predicted 12.69 psi/9.77 min, whereas 9.52% was the experimentally measured validation result at 12.5 psi/10 min. For study [41], numerical optimization predicted 5.053 bar/51.857 min, whereas 40.23% was the experimentally measured validation result at 5 bar/52 min.

**Table 4:** Physicochemical endpoints co-measured with PME, PPO, and POD responses in hydrodynamic cavitation–processed juices.

Juice Matrix/ Target Enzyme(s)	Functionally Relevant Same-Study Physicochemical Endpoint(s)	Linkage Grade/ Principal Attribution Boundary
Orange (Nagpur); PME, POD [31]	Viscosity; total color difference (ΔE)	Indirect/non-specific. Viscosity is relevant to PME-associated physical behavior and ΔE to POD-associated color response, but neither endpoint is enzyme-specific. Recirculation, homogenization, thermal history, and other treatment effects can modify both independently of enzyme inactivation
Orange (Valencia, Brazil); PME [32]	Sedimentation behavior; rheological behavior	Functionally relevant but non-specific. PME and physical-stability endpoints were measured within the same study, but imposed heating and pronounced particle-size reduction/homogenization provide parallel pathways to the observed product response
Tomato; PME [33]	Sedimentation index; viscosity	Functionally relevant but non-specific. PME and physical-stability endpoints were assessed within the same experimental design. Because PME control remained limited while mechanical size reduction and bulk heating occurred concurrently, improved physical behavior cannot be assigned to PME reduction alone
Orange (Valencia, Brazil); PME [34]	Sedimentation/physical-stability behavior	Functionally relevant but non-specific. Physical stability was assessed together with PME response, but prolonged recirculation, particle-size reduction, and increasing bulk temperature provide parallel explanations for the observed stabilization
Huyou turbid juice; PME [35]	Pectin molecular and structural characteristics; pectin rheology; cloud/turbidity stability	Relatively direct. PME, endogenous pectin characteristics, and turbidity stability were assessed within the same experimental system. Pectin characteristics also changed during HC processing; therefore, improved stability cannot be assigned solely to PME reduction
Huyou juice; PME, PPO, POD [36]	Cloud value and turbidity; instrumental color and ΔE	Functionally relevant but non-specific. Cloud/turbidity are functionally relevant to PME and color to PPO/POD, but all three enzymes and multiple matrix properties changed concurrently. The contribution of individual enzymes cannot be separated from matrix restructuring or other treatment effects
Aonla (Indian gooseberry); PPO [37]	Browning index; total color difference (ΔE)	Functionally relevant but non-specific. Browning and color are functionally relevant to PPO and were assessed within the same study. Their response remains treatment-level because substrate availability, oxygen exposure, temperature, matrix changes, and non-enzymatic reactions can also affect these endpoints
Sugarcane + 30 mg/100 mL AA; PPO, POD [38]	Total color difference (ΔE)	Indirect/non-specific. Color was assessed together with PPO and POD responses, but the contributions of the two enzymes cannot be separated and AA directly modifies browning/oxidation chemistry. The correspondence therefore applies to the combined AA + temperature-management + HC treatment
Apple (Delicious Red); PPO [39]	Total color difference (ΔE)	Indirect/non-specific. PPO and color were assessed within the same experimental design, but ΔE is not PPO-specific. The pronounced cloud, sedimentation, and particle-size responses reported in the study represent parallel physical effects and should not be interpreted as PPO-mediated stabilization
Fresh sugarcane; PPO [40]	Browning index; total color difference (ΔE)	Functionally relevant but non-specific. PPO and directly relevant browning/color endpoints were included in the same multiresponse design. Their concurrent response supports functional relevance but does not establish that PPO reduction caused the observed color response
Mixed-fruit juice; POD [41]	Total color difference (ΔE)	Indirect/non-specific. POD and ΔE were included in the same multiresponse optimization. ΔE is not POD-specific and can also reflect pigment degradation, non-enzymatic oxidation, thermal exposure, and matrix-level changes

Note: HC, hydrodynamic cavitation; PME, pectin methylesterase; PPO, polyphenol oxidase; POD, peroxidase; AA, ascorbic acid; ΔE, total color difference. Co-measurement of an enzyme response and a physicochemical endpoint does not establish enzyme-mediated causality. Particle-size reduction, homogenization, thermal reactions, formulation, and other parallel pathways may modify these endpoints independently of enzyme inactivation.

**Table 5:** Experimentally measured quality-retention patterns and treatment burden during hydrodynamic cavitation processing of real juice matrices.

Juice Matrix/ Target Enzyme(s)	Quality-Retention/Burden Evidence	Supported Treatment-Level Interpretation
Orange (Nagpur); PME, POD [31]	At 5 bar/15 min, vitamin C and antioxidant retention were 94.43% and 95.05%; at 13 bar/10 min, they were 85.71% and 93.38%, respectively	The two multiresponse-selected conditions differed appreciably in vitamin C retention, showing that treatment selection involves a quality dimension in addition to enzyme response
Orange (Valencia, Brazil); PME [32]	Vitamin C decreased from 49.78 mg/100 mL in untreated juice to 16.01 mg/100 mL at 70 °C/8 min; the thermal comparator contained 15.16 mg/100 mL	The most severe thermally assisted HC condition imposed a substantial vitamin C burden approaching that of the thermal comparator
Tomato; PME [33]	The optimized HC treatment retained 93% ascorbic acid and 96.6% total phenolics and preserved these bioactives better than thermal processing	Multiresponse selection favored substantial bioactive retention relative to conventional thermal treatment
Orange (Valencia, Brazil); PME [34]	Vitamin C decreased from 33.83 ± 0.15 mg/100 mL in untreated juice to 16.65 ± 0.32 mg/100 mL after 60 min and 15.57 ± 0.13 mg/100 mL after 90 min	Increasing recirculation time was accompanied by a progressive nutritional burden
Huyou juice; PME, PPO, POD [36]	Ascorbic acid decreased with treatment duration, whereas total phenolics increased; sensory quality remained generally more favorable than after thermal pasteurization	Quality dimensions did not change in a uniform direction, so overall treatment quality cannot be represented by a single compositional endpoint
Aonla (Indian gooseberry); PPO [37]	The optimized treatment retained 88.01% vitamin C and approximately 92% antioxidant activity; both declined as pressure and treatment time increased	Increasing severity imposed a measurable bioactive burden, while optimization favored greater quality retention than thermal processing
Sugarcane + 30 mg/100 mL AA; PPO, POD [38]	At the validated 4 °C/30 min condition, total phenolics were 62.40 ± 0.01 mg GAE/100 mL, flavonoids 2.67 ± 0.10 mg QE/mL, and antioxidant activity 83.35 ± 0.10%. These responses declined at greater temperature/time exposure in the factorial design	Favorable bioactive and antioxidant responses were retained within the combined AA + temperature-management + HC process, with greater exposure imposing a larger quality burden
Apple (Delicious Red); PPO [39]	Antioxidant activity decreased after HC relative to untreated juice but remained higher than after thermal processing	HC imposed a measurable antioxidant burden, although smaller than that associated with the thermal comparator
Fresh sugarcane; PPO [40]	At the separately reported 12.5 psi/10 min comparison, vitamin C and antioxidant capacity were 6.35 mg AAE/mL and 34.93 mg GAE/100 mL, respectively, compared with 6.77 mg AAE/mL and 36.45 mg GAE/100 mL in untreated juice; greater treatment severity reduced both	The selected operating region represented a compromise between treatment intensity and nutritional preservation
Mixed-fruit juice; POD [41]	At the experimentally validated 5 bar/52 min condition, vitamin C was 0.378 mg/mL, total phenolic content was 32.22 mg GAE/100 mL, and antioxidant activity was 26.63% DPPH inhibition.	Measured vitamin C, total phenolic content, and DPPH antioxidant activity were higher than after thermal processing, while POD inactivation remained partial.

Note: HC, hydrodynamic cavitation; PME, pectin methylesterase; PPO, polyphenol oxidase; POD, peroxidase; AA, ascorbic acid; GAE, gallic acid equivalents; QE, quercetin equivalents. Quality endpoints describe product-level retention or treatment burden and do not establish that the observed quality response was mediated by enzyme inactivation. Conventional thermal treatments provide process-level context rather than severity-matched controls. For combined treatments, the reported quality response applies to the complete processing package.

**Table 6:** Exact same-condition PPO measurements during refrigerated storage.

Juice Matrix/Study	HC Condition	Storage	PPO Inactivation at Day 0 (%)	PPO Inactivation at End of Storage (%)
Apple [39]	86.18 kPa/10 min	15 d at 4 °C	5.55 ± 0.32	4.80 ± 0.32
Fresh sugarcane [40]	12.5 psi/10 min	14 d at 4 °C	12.8 ± 0.1	7.3 ± 0.6
Fresh sugarcane [40]	15 psi/30 min	14 d at 4 °C	18.7 ± 0.1	9.0 ± 1.4

Note: HC, hydrodynamic cavitation; PPO, polyphenol oxidase. Only exact day-0 and end-of-storage PPO measurements reported for the same HC treatment condition were included. Values are reported as mean ± standard deviation in the source studies. The two fresh-sugarcane trajectories originate from the same publication and therefore do not represent independent study-level replications. No pooled storage estimate was calculated. For fresh sugarcane, the day-0 values are those reported directly as the baselines of the corresponding storage series [40]. The 12.8 ± 0.1% day-0 value at 12.5 psi/10 min therefore represents the storage-series baseline and should not be interpreted as the same measurement as the 9.52% optimization-validation result reported elsewhere in the source.

**Table 7:** Source-reported baseline matrix descriptors relevant to cross-matrix interpretation of HC enzyme responses.

Juice/Study	Acidity (pH; TA)	TSS/Formulation	Physical Matrix Descriptor	Initial Enzyme Basis
Orange [31]	3.78; 1.2% citric acid	11.7 °Brix	NR	PME 4.79 meq H^+^ mL^−1^ min^−1^; POD 3.19 ΔOD/mL
Orange [32]	3.94; NR	9.0 °Brix	D3,2 98.7 µm; D4,3 565 µm	PME 0.00201 meq H^+^ mL^−1^ min^−1^
Tomato [33]	4.40; 0.260%	5.0 °Brix	NR	PME 2.57 meq H^+^ mL^−1^ min^−1^
Orange [34]	3.8; NR	8.9 °Brix	Particle size 17.1 µm	PME 0.001837 meq H^+^ mL^−1^ min^−1^
Huyou turbid juice [35]	NR	NR	Cloud value 37.08%; pectin fractions characterized	PME = 100% reference
Huyou juice [36]	3.45; 2.11%	14.33 °Brix	Cloud value 37.08%; turbidity 0.15	PME, PPO, and POD measured
Aonla [37]	2.58; 1.68%	12.10 °Brix	Cloud value 0.114	PPO = 100% reference
AA-treated sugarcane [38]	5.23 to 4.90 after AA addition	30 mg AA/100 mL before HC	NR	PPO and POD measured after AA addition
Apple [39]	4.03; 0.281%	11.52 °Brix	Cloud value 0.283; particle size 8934 nm	PPO = untreated reference
Sugarcane [40]	5.19; 0.396% citric acid	19.6 °Brix	NR	PPO = untreated reference
Mixed-fruit juice [41]	3.45; 0.592% citric acid	10.4 °Brix; 60/25/15% mandarin/ pineapple/ watermelon	NR	POD = untreated reference

Note: NR, no directly usable baseline value reported; TA, titratable acidity; TSS, total soluble solids; AA, ascorbic acid. Values are source-reported baseline descriptors. TA values were not harmonized across acid equivalents, and TSS was not treated as a direct measure of sugar concentration. A harmonized sugar-to-acid ratio was therefore not calculated. Enzyme activities were not converted across assay or reporting scales.

**Table 8:** Three priority knowledge gaps, research directions, and validation methods for hydrodynamic cavitation in juice processing.

Key Knowledge Gap	Priority Research Direction	Feasible Validation Method
Incomplete separation of cavitation-associated, thermal, and hydraulic effects	Improve attribution of treatment responses to distinct process contributions	Compare the full HC treatment with a closely matched non-cavitating hydraulic comparator and, where relevant, a thermal-history-matched comparator, using the same matrix, enzyme assay, and product endpoints
Limited validation across matrices, reactors, and scale	Test transferability without assuming equivalence of nominal operating settings	Re-characterize the hydraulic and thermal process state under transferred conditions and revalidate enzyme, physicochemical, and quality responses against predefined performance criteria
Sparse paired enzyme–function–quality data during storage	Define storage trajectories and evaluate predictive models only when supported by longitudinal data	Perform repeated paired measurements at prespecified storage times under defined packaging and storage conditions and validate any predictive model using independent data

## Data Availability

No new data were created or analyzed in this study. Data sharing is not applicable to this article.
